# Agreement between routine electronic hospital discharge and Scottish Stroke Care Audit (SSCA) data in identifying stroke in the Scottish population

**DOI:** 10.1186/s12913-015-1244-y

**Published:** 2015-12-30

**Authors:** Melanie Turner, Mark Barber, Hazel Dodds, Martin Dennis, Peter Langhorne, Mary-Joan Macleod, on behalf of the Scottish Stroke Care Audit

**Affiliations:** Division of Applied Medicine, Department of Medicine and Therapeutics, Polwarth Building, Foresterhill, University of Aberdeen, Aberdeen, UK; NHS Lanarkshire Stroke MCN, Stroke Unit, Monklands Hospital, Monkscourt Avenue, Airdrie, UK; Information Services Division, NHS National Services Scotland, Edinburgh, UK; Centre for Clinical Brain Sciences, University of Edinburgh, Royal Infirmary of Edinburgh, Edinburgh, UK; Institute of Cardiovascular and Medical Sciences, University of Glasgow, Royal Infirmary, Glasgow, UK

**Keywords:** Data validity, Stroke, Admission

## Abstract

**Background:**

In Scotland all non-obstetric, non-psychiatric acute inpatient and day case stays are recorded by an administrative hospital discharge database, the Scottish Morbidity Record (SMR01). The Scottish Stroke Care Audit (SSCA) collects data from all hospitals managing acute stroke in Scotland to support and improve quality of stroke care. The aim was to assess whether there were discrepancies between these data sources for admissions from 2010 to 2011.

**Methods:**

Records were matched when admission dates from the two data sources were within two days of each other and if an International Classification of Diseases (ICD) code of I61, I63, I64, or G45 was in the primary or secondary diagnosis field on SMR01. We also carried out a linkage analysis followed by a case-note review within one hospital in Scotland.

**Results:**

There were a total of 22 416 entries on SSCA and 22 200 entries on SMR01. The concordance between SSCA and SMR01 was 16 823. SSCA contained 5593 strokes that were not present in SMR01, whereas SMR01 contained 185 strokes that were not present in SSCA. In the case-note review the concordance was 531, with SSCA containing 157 strokes that were not present in SMR01 and SMR01 containing 32 strokes that were not present in SSCA.

**Conclusions:**

When identifying strokes, hospital administrative discharge databases should be used with caution. Our results demonstrate that SSCA most accurately represents the number of strokes occurring in Scotland. This resource is useful for determining the provision of adequate patient care, stroke services and resources, and as a tool for research.

## Background

Stroke is the third most common cause of mortality and the commonest cause of severe physical disability in Scotland [1]. There are approximately 13 000 people having a stroke each year with stroke patients occupying 7 % of all National Health Services (NHS) beds and their health care costs accounting for approximately 5 % of the entire Scottish NHS budget [2]. To provide the best possible patient care and stroke services, correctly identifying patients affected by stroke is of utmost importance and allows for the appropriate resource allocation [3].

**Fig. 1:**
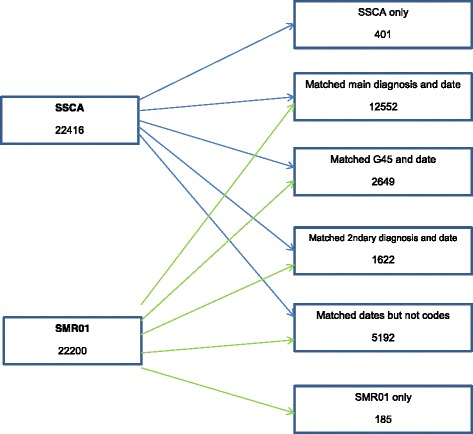
Flow diagram

Administrative hospital discharge coding data are currently collected by most Western countries in some form. These are seen as a useful tool for stroke research as they are anonymous, inexpensive, easily retrievable and available in an electronic format. Despite their advantages, there are limitations; assigning of diagnosis codes from limited information, data acquisition is retrospective, and no information on patient care is provided. The reliability of the recorded diagnoses has been questioned and the accuracy between administrative coding data and medical records has been scrutinised in different settings [4–7].

In Scotland, administrative hospital discharge data is used to determine the level of NHS resource needed for a specific condition. It is therefore important to determine if this contains an accurate account of stroke occurrence. We aimed to assess the discrepancies between a Scotland-wide stroke audit used to assess performance and an administrative hospital discharge database collected during 2010-2011.

## Methods

### Scottish Stroke Care Audit

The Scottish Stroke Care Audit (SSCA) was established in 2002 to monitor performance against guideline based Clinical Standards [8]. It is resourced by Information Services Division (ISD) of National Services Scotland (part of NHS Scotland). Ascertainment and input of stroke cases in the SSCA is a process carried out by trained but non-specialist audit co-ordinators supported by clinicians and based in stroke units within hospitals. The audit collects information about stroke care in hospitals managing acute stroke in Scotland and has provided full national coverage of all hospitals since 2005. The data set covers demographic factors, stroke pathway data, outcome predictors, antithrombotic drug use on admission, medication on discharge, brain imaging and stroke classification.

### Hospital discharge database

Scottish Morbidity Records 01 (SMR01) forms an episode-based record for all non-psychiatric, non-obstetric acute hospital admissions in Scotland. This is also resourced by ISD of National Services Scotland. A record is formed when a patient is discharged from hospital, has a change of consultant, or is transferred to another hospital or department. The tenth revision of the International Classification of Diseases (ICD-10) is used to assign codes to diagnoses. Approximately one million records are created each year by clerical staff using clinical information to assign a primary and up to five secondary diagnoses. The data are widely used within the NHS and the Scottish Government. Data Quality Assurance Assessments for SMR01 are performed periodically and suggest that 88 % of the sample of records had the correct codes [3].

### Linkage analysis and agreement between the SSCA and SMR01

Data for 2010-2011 were requested from ISD in Scotland, where a data analyst linked records between the two data sources. Records were considered to be matched when the dates of admission in an individual person from the two data sources were within two days of each other and if an ICD code of I61 (Intracerebral haemorrhage), I63 (Cerebral Infarction) or I64 (Stroke, not specified as haemorrhage or infarction) was in the primary diagnosis field on SMR01.

In contrast to previous linkage work in Scotland [9] we also investigated the inclusion of G45 (Transient Ischaemic Attack (TIA)) as a primary diagnosis code and all four codes, I61, I63, I64 and G45 as secondary diagnoses within SMR01.

To investigate discrepancies in more detail we also carried out a linkage analysis followed by a case-note review within one hospital in Scotland for data entered between 1^st^ Jan 2010-31^st^ October 2010. A data analyst within NHS Grampian Health Intelligence produced a linked dataset and a research fellow carried out the case-note review.

### Ethics approval

While individual patient consent was not obtained, the SSCA works within the “NHS Code of Practice on Protecting Patient Confidentiality” [10] which incorporates the requirements of statute and common law including the Data Protection Act, the Human Rights Act and the Adults with Incapacity (Scotland) Act. The study was approved by Scotland A Research Ethics Committee, Ref. No. = 10/MRE00/76 and the Privacy Advisory Committee of ISD, NHS Scotland, Ref 76/11.

## Results

### Scotland-wide analysis

There were a total of 22 416 entries on SSCA, and 22 200 entries on SMR01 which contained a relevant ICD code in either the primary or secondary diagnosis fields.

12,552 records were found in both SSCA and SMR01 when matching on both date of admission and a main diagnosis code of I61, I63, and I64. Including G45 as a main diagnosis code, increased this to 15 201, and including all four codes in secondary diagnoses fields increased this to 16 823.

5593 records were only present in SSCA. Following cross-referencing with SMR01, 5192 matched with SMR01 on admission date, but had none of the four codes in any of the diagnoses fields. 401 had no match with admission date or diagnoses codes (see Figure 1).

The five most common main diagnoses in the SMR01 records that matched on dates with SSCA but not diagnoses codes, were R298 (other and unspecified symptoms and signs involving the nervous and musculoskeletal system), R55 (syncope and collapse), G819 (unspecified hemiplegia), R51 (headache), and I679 (unspecified cerebrovascular disease). In total across 5192 records there were a total of 614 different ICD-10 codes in the main diagnosis field.

185 of records appeared on only SMR01, of these 158 had one of the four codes in the main diagnosis field.

The results from this analysis suggest that from 1^st^ January 2010 to 31^st^ December 2011 there was concordance between the two datasets of 16 823 strokes. SSCA contained 5593 strokes that were not present in SMR01, and SMR01 contained 185 strokes that were not present in SSCA. Together the SSCA and SMR01 datasets recorded a total of 22 601 strokes in Scotland between 2010 and 2011.

### Individual hospital analysis

There were a total of 664 entries on SSCA, and 492 entries on SMR01 which contained a relevant ICD code in either the primary or secondary diagnosis fields.

Initial matching on admission date and a main diagnosis of stroke showed concordance of 436 entries in both the SSCA and SMR01, while 228 were present only in SSCA and 56 only in SMR01.

Repeat analysis, including all entries with a main diagnosis of stroke or TIA, resulted in a further 54 entries being matched in both SSCA and SMR01. Of the remaining entries only on SMR01, following case-note review a small number of these could be discounted as they had already been entered in SSCA and then excluded because they did not have a diagnosis of stroke. This resulted in 496 entries in both SSCA and SMR01, with 171 only in SSCA and 53 only in SMR01.

Following case-note review of the entries only on SMR01, 29 were confirmed to have had a stroke diagnosis and should have been in the SSCA and the remaining 19, which had been coded with stroke as the main diagnosis, had another diagnosis following case note review.

Of the entries only in SSCA, reasons for not appearing in SMR01 included, being missed due to different admission hospitals or having stroke as a secondary diagnosis code or no mention of stroke in the diagnoses codes. For a high number of entries the most common primary and secondary diagnoses codes were I67 (other cerebrovascular diseases), I62 (other non-traumatic intracranial haemorrhage), J69 (pneumonitis due to solids and liquids), R55 (syncope and collapse), R42 (dizziness and giddiness), R51 (headache), G83 (other paralytic symptoms), S72 (fracture of the femur) and S09 (unspecified injury to head).

Following this further investigation the final figures showed there was concordance of 531 between the SSCA and SMR01, with 157 only in SSCA, and 32 only in SMR01, from 1^st^ January 2010 to 31^st^ October 2010. Together the SSCA and SMR01 datasets recorded a total of 720 strokes.

## Discussion

Our data show that there was concordance of 16 823 strokes between SSCA and SMR01 in Scotland between 1^st^ January 2010 to 31^st^ December 2011. SSCA identified more strokes admitted to hospital in Scotland compared with administrative coding data for SMR01.

This was an increase in concordance from previous linkage data carried out in Lothian, Scotland. Covering 2000-2005 this showed that 12 859 records were found both in SMR01 and SSCA, 5764 in SSCA only, and 7795 in SMR01 only [9]. From 2006-2009, 18 089 records were found in both SMR01 and SSCA, 8318 in SSCA only and 9902 in SMR01 only. These increases may be due to training of coding staff and an improvement in coding on hospital discharge letters. In addition the inclusion of G45 as a main diagnosis code and including all four codes in secondary diagnoses fields in this study will have contributed.

Problems in primary diagnoses coding errors was the main reason for SMR01 not identifying all strokes. There could also be discrepancies with admission dates where the hospital admission dates were outside the +/- two days matching criteria. Possible reasons for stroke diagnosis being missed by SSCA could be coding errors in stroke diagnosis, over-riding diagnoses such as infection or myocardial infarction, admission with a complication such as fracture, stroke occurring in hospital, death soon after admission, hospital transfer or quick discharge.

SMR01 entries are carried out by coders who have no direct link to clinical teams but rely on hospital discharge letters, thus the quality of the data is dependent on both the quality of documentation and the experience and expertise of the coder. In comparison the ascertainment of stroke cases in the SSCA is a process carried out using detailed data collection forms by trained but non-specialist audit co-ordinators supported by clinicians and based in stroke units within hospitals. Quality assurance is carried out regularly by ISD staff.

Records from both SSCA and SMR01 are limited to patients that are admitted to hospital having suffered a stroke. Therefore, people who have a stroke or TIA but are never admitted to hospital will be missed by both sources. Any future work in this area could use both further sources of data (such as out-of-hospital stroke mortality data) and the capture-recapture technique to provide more reliable estimates of the total number of stroke occurring in the population.

In recent studies investigating hospital discharge data and medical records in France, the main factors associated with lack of correlation were misdiagnosis of stroke, lack of precision regarding the cause of stroke, and errors in coding [6, 7]. These studies concluded that administrative coding data within France cannot be considered as the sole means of identifying stroke occurrence rates.

When using hospital administrative discharge databases to identify strokes, we agree that this should be done with caution. Although secondary prevention is similar for both TIA and stroke, basing economic models on datasets which do not distinguish between transient and disabling events, for example, may result in inaccurate cost calculations.

## Conclusions

Our results show the robustness of the SSCA dataset and demonstrate that it more accurately represents the number of strokes admitted to hospital in Scotland compared with SMR01. There may, however,be variation across individual NHS health boards. In addition, SSCA collects information on risk factors, stroke pathway data and outcome predictors which are not available from administrative coding data. This represents a valuable resource to underpin investigation of the provision of adequate patient care, stroke services and resources, and also as a tool for research [11, 12].

## Abbreviations

SSCA: Scottish Stroke Care Audit
SMR01: Scottish Morbidity Record 01
ICD: International Classification of Diseases
NHS: National Health Service
ISD: Information Statistics Division
TIA: Transient Ischaemic Attack
